# Pathological Threshold of Patellar Tendon-Trochlear Groove Distance Using Magnetic Resonance Image in Patients with Patellar Instability

**DOI:** 10.1055/s-0045-1806817

**Published:** 2025-06-23

**Authors:** Rubens Rosso Nadal, Vinícius Canello Kuhn, Alexandre Codevilla Teixeira, Eduardo Bervian Júnior, Osmar Valadão Lopes Júnior

**Affiliations:** 1 Instituto de Ortopedia e Traumatologia de Passo Fundo, Passo Fundo, RS, Brazil; 2 Department of Orthopedics and Traumatology, Hospital São Vicente de Paulo, Passo Fundo, RS, Brazil; 3 Department of Musculoskeletal Radiology, Clínica Kozma, Passo Fundo, RS, Brazil

**Keywords:** joint instability, knee, magnetic resonance imaging, patellar ligament, patellofemoral joint, articulação patelofemoral, imagem por ressonância magnética, instabilidade articular, joelho, ligamento patelar

## Abstract

**Objective:**

To evaluate and compare the values of the patellar tendon-trochlear groove (PT-TG) distance in individuals with and without patellar instability. Additionally, we aimed to define a cut-off value for the pathological limit of the PT-TG through nuclear magnetic resonance imaging (MRI) in patients with patellar instability.

**Methods:**

The PT-TG distance of 52 knees was measured in 48 individuals with objective patellar instability (instability group) by MRI. These measurements were compared with those made in 50 knees of 44 individuals without a history of patellar instability (control group).

**Results:**

The PT-TG distance in the instability group (20.6 ± 5.0 mm) was greater than in the control group (11.8 ± 3.4 mm; *p* < 0.001). A value of 15.5 mm was determined as the pathological limit, with an accuracy of 81.4%, in MRI exams.

**Conclusion:**

Individuals with patellar instability have statistically higher measurements of PT-TG when compared with patients without it. Therefore, values higher than 15.5 mm for PT-TG seen in MRI exams represent a pathological lateralization force of the extensor mechanism related to patellar instability.

## Resumo

**Objetivo:**

Avaliar e comparar a distância tendão patelar–sulco troclear (TP-ST) em indivíduos com e sem instabilidade patelar. Um segundo objetivo foi definir um limiar patológico de TP-ST por meio de ressonância magnética (RM) nuclear em pacientes com instabilidade patelar.

**Métodos:**

A distância TP-ST de 52 joelhos foi medida em 48 indivíduos com instabilidade patelar objetiva (grupo instabilidade) por RM. Essas medidas foram comparadas àquelas feitas em 50 joelhos de 44 indivíduos sem histórico de instabilidade patelar (grupo controle).

**Resultados:**

A distância TP-ST no grupo instabilidade (20,6 ± 5,0 mm) foi maior do que no grupo controle (11,8 ± 3,4 mm; *p* < 0,001). O valor de 15,5 mm foi determinado como limiar patológico, com acurácia de 81,4%, em exames de RM.

**Conclusão:**

Indivíduos com instabilidade patelar apresentam medidas estatisticamente maiores de TP-ST em comparação a pacientes sem instabilidade. Portanto, valores de TP-ST superiores a 15,5 mm à RM representam uma força de lateralização patológica do mecanismo extensor relacionada à instabilidade patelar.

## Introduction

Lateral patellar instability is a pathology with an incidence ranging between 7 and 43 cases per 100 thousand inhabitants. 1 2 Anatomical risk factors are described as the main causes for this condition: trochlear dysplasia, increased tibial tuberosity-trochlear groove (TT-TG) distance, patellar tilt, and patella alta. 3 4

The TT-TG distance is the distance between the deepest bone point of the femoral trochlea (trochlear groove, TG) and the most anterior point of the anterior tibial tuberosity (ATT). It is measured in a computed tomography (CT) scan of the knee, and patients with values equal to or greater than 20 mm, in cases of recurrent objective lateral patellar dislocations, are referred for ATT osteotomy for realignment. 4 5

Complementary imaging exams are important for identifying risks factors to patellar instability and, thus, for choosing which procedures should be performed. A CT scan of the knee is important to evaluate the TT-TG distance according to the Lyon Protocol. 4

Usually, nuclear magnetic resonance imaging (MRI) of the knee is useful in the evaluation of articular cartilage, ligaments, and the meniscus. 6 Although a CT scan is considered the exam of choice for the measurement of the TT-TG distance, authors have demonstrated that MRI can also be used to measure it with excellent inter and intraobserver agreement. 6 7 8 Despite this, it is important to note that the values measured by both methods are not interchangeable. Magnetic resonance imaging measurements tend to be lower than those of a CT scan. 7 The values obtained by MRI are more anatomical compared with the ones by CT and would represent, in a more reliable way, the biomechanical vector that act on patellar instability. 8 The CT scan request would also be dispensable, reducing costs and patient exposure to radiation.

The present study aimed to evaluate the distance measurement between the center of the patellar tendon and the cartilage of the femoral trochlear groove (PT-TG) in MRI exams of the knees in patients with objective patellar instability and to compare them with the values of subjects with no patellar instability, as well as to define the pathological cut-off point of this distance measurement.

## Materials and Methods

In a case-control study, we have analyzed 102 knee MRI scans in 92 individuals, selected through simple random sampling from electronic medical records and subsequent analysis in an image bank.

The PT-TG distance of 52 knees was measured in 48 individuals with a documented history of objective patellar instability (instability group). These measurements were compared with those of 50 knees of 44 individuals who underwent a knee MRI without having a clinical history of patellar instability (control group).

The inclusion criteria for the instability group were patients with objective lateral patellar instability 9 documented by history and physical examination in the medical records of our database, in addition to indirect sights on the MRI. 10 11 12 13 These indirect sights could be contusion or osteochondral lesion of the lateral femoral condyle or medial facet of the patella, injury to the medial patellofemoral ligament, injury to the medial retinaculum in its patellar insertions or middle substance, laceration of the distal belly of the vastus medialis oblique muscle, and patellar subluxation and tilt.

From the instability group, we excluded patients with previous knee operations, who had a history of trauma (except for patellar dislocation) and presented moderate-to-severe degenerative pathologies of the joint. In the control group, we placed patients who underwent knee MRI for suspected intra-articular ligament injuries, meniscal injuries, and pain, resulting from no history of dislocation or previous patellofemoral symptoms.

**Fig. 1:**
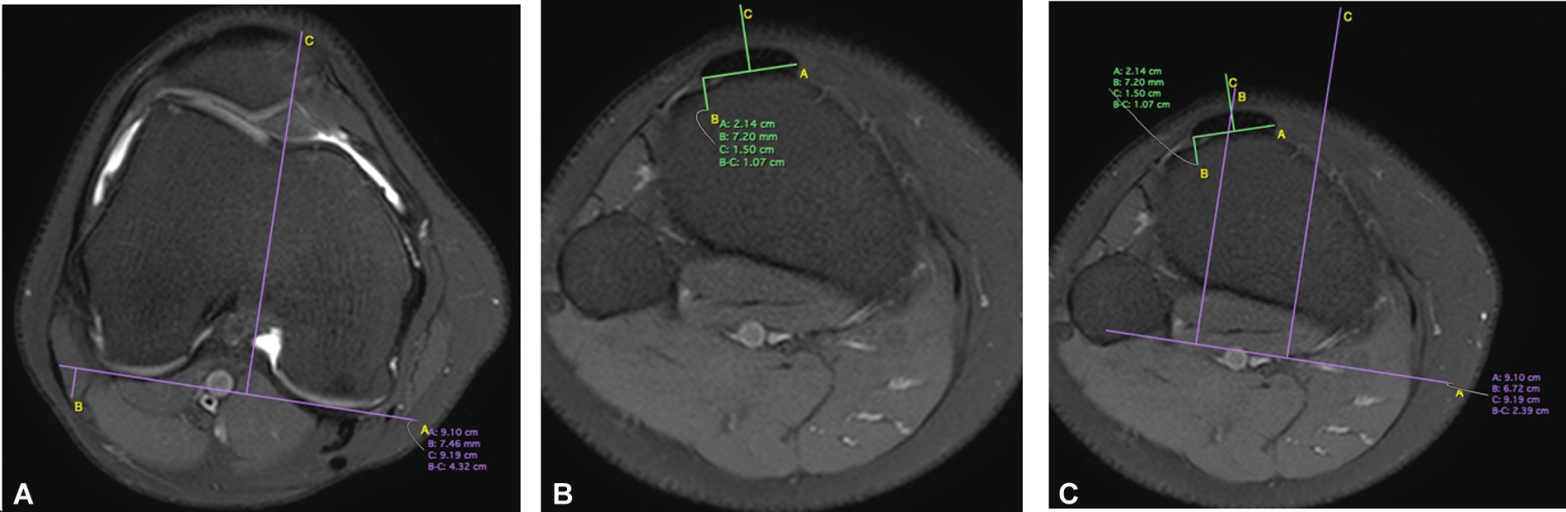
Measurement of the patellar tendon-trochlear groove (PT-TG) distance. ( **A** ) The deepest trochlea point, TG; ( **B** ) the center of the patellar tendon, PT; and ( **C** ) the distance between these two overlapping points, PT-TG.

Magnetic resonance imaging was performed with the patient lying supine, the limb in neutral rotation and full extension without quadriceps contraction. To obtain the measurement of the PT-TG distance, the axial T2-weighted MRI cut was used, and the following parameters, as described by Schoettle et al., 8 were observed. The first craniocaudal image that completely demonstrates the entire trochlear cartilage was used to determine the TG, using the cartilaginous edges of the posterior femoral condyles as a reference. The PT is defined in the distally subsequent images in its insertion on anterior tibial tubercle (ATT). The previous TG images were then transferred to the current image, which corresponds to the overlap between the center of the PT and the deepest point of the TG. Thus, the PT-TG is measured in millimeters ( Fig. 1 ).

The measurements were performed by two orthopedists and a radiologist specialized in musculoskeletal MRI using the OsiriX DICOM Viewer (Pixmeo SARL, Geneva, Switzerland) software. Each MRI was evaluated by the three evaluators who repeated their evaluations with a minimum interval of two weeks between measurements. At no time did the evaluators have access to which group the individuals belonged to, the values found by their peers, and their previous measurement.

The exams were performed between April 2014 and September 2019, and the study was approved by our institution's Ethics Committee under number CAAE: 36188820.3.0000.5342.

### Statistical Analysis

Quantitative variables were described by the mean and standard deviation, and categorical variables by absolute and relative frequencies. To compare means between the groups, the Student's t-test was applied. When comparing proportions, the Pearsons Chi-squared test was used. To assess the intra- and inter-examiner agreement, the Student's *t* -test for paired samples and the intraclass correlation coefficient (ICC) were applied. The receiver operating characteristic (ROC) curve was used to determine the best cut-off point for the PT-TG measurement in detecting knee instability. The level of significance adopted was 5% ( *p* < 0.05), and the analyses were performed using the IBM SPSS Statistics for Windows (IBM Corp., Armonk, NY, USA) software, version 21.0.

## Results

Mean age was similar in both groups, with 23.3 ± 8.3 years in the instability group and 25.4 ± 6.4 years in the control group. Regarding the side, 53% and 48% of the measurements were taken on the right knee of the instability and control groups, respectively. As for gender, there was a statistically significant difference between the groups. Female patients accounted for 65.4% of the instability group and 22% of the control group.

**Fig. 2:**
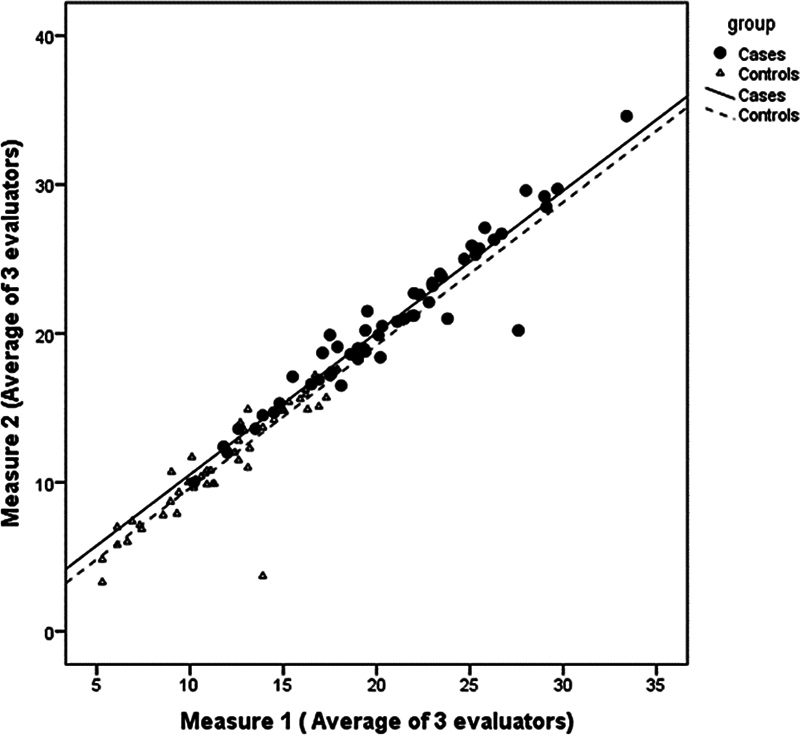
Evaluation of the intra-examiner agreement.

Considering the intra-examiner agreement, a good relationship is observed between the two measurements made by the three examiners. The ICC was 0.99 in the instability group and 0.98 in the control group. Fig. 2 shows the analyzed data.

**Fig. 3:**
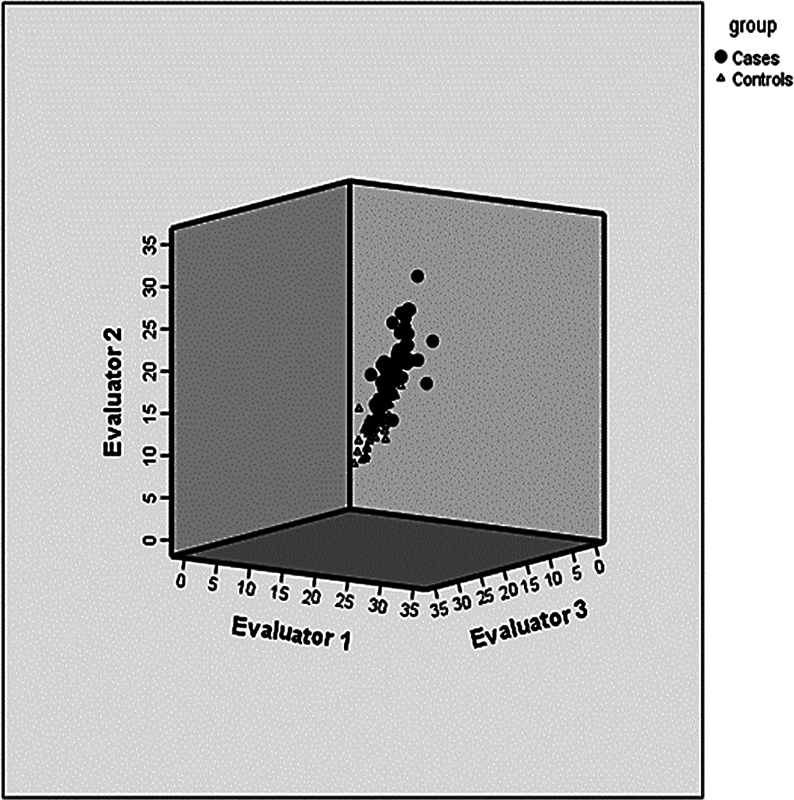
Evaluation of the inter-examiner agreement.

There was no significant difference in the values found by the examiners in the Instability group. In the control group, examiner 3 had slightly lower averages than examiner 2. Nevertheless, the ICC was 0.98 in both groups, showing good inter-examiner agreement ( Fig. 3 ).

**Fig. 4:**
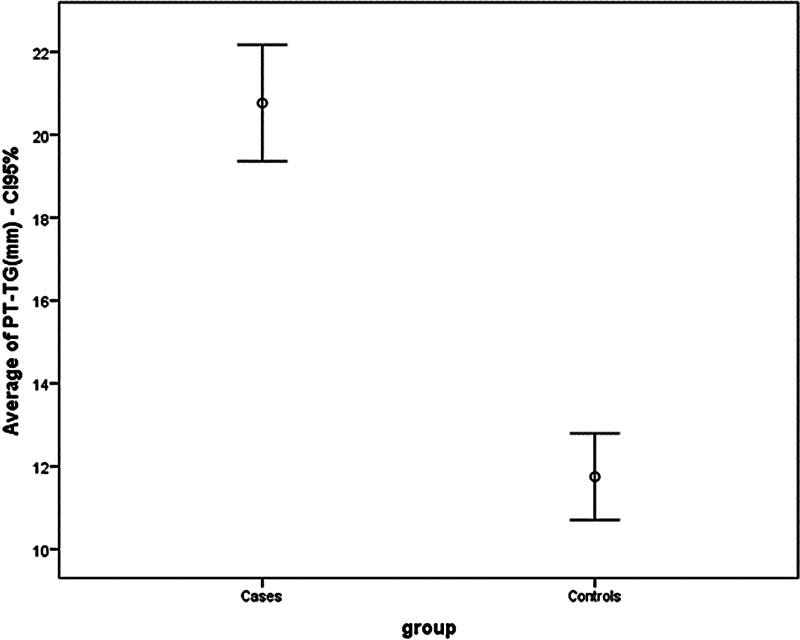
Comparison of the TT-TG distance measurement between groups.

Objectively, in terms of the value of PT-TG, a higher average was observed in the instability group when compared with the control group. In the first one, the average was 20.6 ± 5.0 mm, while the average for the second group was 11.8 mm ± 3.4 mm ( *p* < 0.001). The comparison is illustrated in Fig. 4 .

**Fig. 5:**
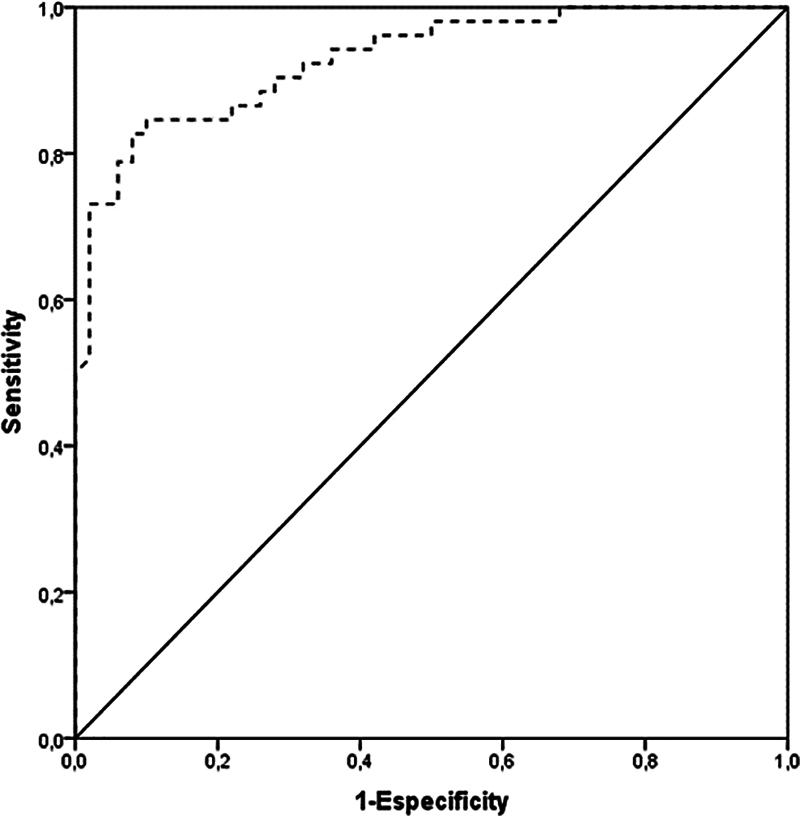
Receiver operating characteristic curve to determine the best cut-off point for measuring the PT-TG distance in patellar instability screening.

Finally, the pathological limit defined as a new cut-off point for measuring PT-TG in MRI scans is 15.5 mm. This was determined by the ROC, reaching a sensitivity of 82.7%, specificity of 80.0%, positive predictive value of 81.1%, negative predictive value of 81.6%, and accuracy of 81.4%. This cutt-off point is consistent as a tracking variable, as shown in Fig. 5 .

## Discussion

The main findings of the present study are that the individuals with lateral patellar instability have a greater measure of PT-TG when compared with patients without it. Besides, the value of 15.5 mm for PT-TG in MRI exams represents a pathological threshold related to lateral patellar instability.

The lateralized force vector of the knee extensor mechanism is recognized as one of the main factors for patellar instability, and CT scans may still be considered the gold standard to assess it. However, it has become debatable since more specific and sensitive exams have become widely accessible. In the current context, MRI has enormous importance and should be used for diagnoses and surgical planning. It allows the evaluation of the femoral and patellar articular cartilage, the precise identification of the insertion of the patellar tendon, and the integrity of the patellofemoral ligaments. In addition, radiation exposure and the additional cost of CT may be avoided.

The definition of a pathological limit of 15.5 mm in the measurement of PT-TG in MRI scans is an important factor for considering the necessity of ATT osteotomy. Thus, MRI should be considered the only necessary complementary examination, in addition to knee radiography for diagnosis and surgical planning of patellofemoral instability.

With greater availability of detailed imaging examination, as in using MRI, quality was added to preoperative diagnosis. When considering soft parts and joint surfaces, MRI enables for a superior definition than the one provided by a CT scan. Even though the tomographic measure is the gold standard for defining the TT-TG distance, studies showed an interobserver confidence lower than 60% in the measurements made by this method. The greatest difficulty faced was determining the deepest point of the TG, especially in excessively dysplastic knee trochlea. 14

Thus, corroborating with previous studies, 8 15 it is possible to find excellent rates of inter- and intra-observer reliability for PT-TG measurement in knees with previous dislocation or without instability with ICCs as high as 0.98 and 0.99, respectively. This measurement is believed to be more physiological in relation to the bone reference points of a CT, since it truly represents the reference site where the forces responsible for aligning the knee extensor mechanism act. 8 16

According to Camp et al., 7 when analyzing the TT-TG and PT-TG distances in knees with patellofemoral instability using CT and MRI scans, both methods have excellent interobserver reliability. The values of PT-TG distance in MRI were constantly lower, with a bigger difference in cases in which the TT–TG distance measurement in CT was greater than 20 mm in dysplastic knees. Therefore, these results suggest that the measurement of PT-TG distance with MRI is valid, but the values of the two imaging methods are not interchangeable in patients with instability.

Pandit et al., 6 in a study with clinically and arthroscopically normal knee joints, found mean values of 10 ± 1 mm with MRI, and these were lower than what was proposed by Dejour 4 for asymptomatic knees when measured by CT. Similarly, Thakkar et al. 17 observed lower mean values in MRI when compared with CT for knees without instability.

In relation to knees with patellofemoral instability, Skelley et al. 15 found values of 18.2 mm in MRI, again lower than those found in the measurements by CT in previous studies. This pattern followed in later studies, 18 19 20 that is, the measurement of both unstable and unaffected knees of PT-TG distance by MRI showed lower values when compared with the TT-TG distance measured by CT.

Few studies have compared knees with and without instability regarding PT-TG distance measured with MRI; thus, it is necessary to validate a new pathological limit for this variable in this type of exam. The present study found a cut-off point of 15.5 mm, and with high sensitivity and specificity, this value has proved to be a consistent tool for a tracking variable.

In the present study, the groups differed in their gender representation. However, it is important to note that the authors did not consider gender at the time of sample selection and randomization because there is no difference between genders in the mean values for the TT-TG and PT-TG distance measurements described in the literature. 6 21

It is noteworthy that this is a case-control study that used exams that had already been performed. The protocol followed for positioning the patient for knee MRI was the one used in most institutions: the limb in neutral rotation and full extension without quadriceps contraction. Moreover, even though there was a randomization of controls, there were patients who had undergone image examination due to some previous complaints unrelated to the patellofemoral joint; hence, they are not images of completely asymptomatic knee joints.

Individuals with lateral patellar instability have statistically greater measurement of the PT-TG distance than patients without it. Moreover, values greater than 15.5 mm for PT-TG distance in MRI exams represent a pathological lateralization force of the extensor mechanism, which is related to patellar instability.
